# The alarmins S100A8 and S100A9 mediate acute pain in experimental synovitis

**DOI:** 10.1186/s13075-020-02295-9

**Published:** 2020-08-27

**Authors:** Arjen B. Blom, Martijn H. van den Bosch, Esmeralda N. Blaney Davidson, Johannes Roth, Thomas Vogl, Fons A. van de Loo, Marije Koenders, Peter M. van der Kraan, Edwin J. Geven, Peter L. van Lent

**Affiliations:** 1 Experimental Rheumatology, Radboud university medical center, Geert Grooteplein 28, 6525 GA Nijmegen, The Netherlands; 2 Institute of Immunology, University of Münster, Münster, Germany

**Keywords:** Synovitis, Pain, Nociception, Alarmins, S100A8, S100A9, Sensitization, Gait analysis, Incapacitance, TLR4

## Abstract

**Background:**

Synovitis-associated pain is mediated by inflammatory factors that may include S100A8/9, which is able to stimulate nociceptive neurons via Toll-like receptor 4. In this study, we investigated the role of S100A9 in pain response during acute synovitis.

**Methods:**

Acute synovitis was induced by streptococcal cell wall (SCW) injection in the knee joint of C57Bl/6 (WT) and *S100A9*^−/−^ mice. The expression of S100A8/A9 was determined in serum and synovium by ELISA and immunohistochemistry. Inflammation was investigated by ^99m^Tc accumulation, synovial cytokine release, and histology at days 1, 2, and 7. To assess pain, weight distribution, gait analysis, and mechanical allodynia were monitored. Activation markers in afferent neurons were determined by qPCR and immunohistochemistry in the dorsal root ganglia (DRG). Differences between groups were tested using a one-way or two-way analysis of variance (ANOVA). Differences in histology were tested with a non-parametric Mann–Whitney *U* test. *p* values lower than 0.05 were considered significant.

**Results:**

Intra-articular SCW injection resulted in increased synovial expression and serum levels of S100A8/A9 at day 1. These increased levels, however, did not contribute to the development of inflammation, since this was equal in *S100A9*^*−/−*^ mice. WT mice showed a significantly decreased percentage of weight bearing on the SCW hind paw on day 1, while *S100A9*^*−/−*^ mice showed no reduction. Gait analysis showed increased “limping” behavior in WT, but not *S100A9*^*−/−*^ mice. Mechanical allodynia was observed but not different between WT and *S100A9*^*−/−*^ when measuring paw withdrawal threshold. The gene expression of neuron activation markers NAV1.7, ATF3, and GAP43 in DRG was significantly increased in arthritic WT mice at day 1 but not in *S100A9*^*−/−*^ mice.

**Conclusions:**

S100A8/9, released from the synovium upon inflammation, is an important mediator of pain response in the knee during the acute phase of inflammation.

## Background

Synovitis is a hallmark of rheumatoid arthritis (RA) and the majority of osteoarthritis (OA) patients. Both diseases have a huge socio-economical impact on society [1]. Pain, the major clinical symptom for the RA and OA patients, is of unknown origin and correlates poorly with radiographic disease scores, and very often, treatment responses are low. Therefore, there is an urgent need to clarify the mechanisms behind pain perception in rheumatic diseases. It is thought that part of the pain perception in RA and OA is related to mediators of synovitis [2–4].

In several studies, we demonstrated that S100A8/9 are involved in inflammation and subsequent structural joint pathology, in humans and experimental RA and OA. S100A8 and S100A9 are proteins that form heterodimers and are abundantly expressed and released by neutrophils, monocytes, and activated macrophages. S100A8/9 heterodimers have been shown to signal via Toll-like receptor 4 (TLR4) and are important regulators of the innate inflammatory response [5]. We demonstrated significantly higher levels of S100A8/9 levels in early OA patients that show progression of disease regarding Kellgren and Lawrence score [6], and pathology in experimental RA and OA strongly depends on S100A8/9 [6, 7].

Since S100A8/9 can mediate synovitis in both RA and OA models, it is difficult to investigate whether S100A8/9 plays a direct role in pain perception. TLR4 has been implicated in pain perception, is expressed by afferent sensory neurons, and may play a role in local peripheral nociception [8, 9]. A role for S100A8 was demonstrated in the activation of these neurons, leading to the production of MCP-1 and the local influx of monocytes that produce S100A8 in the dorsal root ganglion (DRG) [10]. Therefore, S100A8/9 may not only be involved in local nociception but also in sensitization by monocyte influx or local MCP-1 production in the DRG. Sensitization leads to increased pain perception and is thought to be involved in chronic pain that occurs in OA [11].

Activation of the peripheral C- and Aδ-fibers leads to pain sensation [12]. Upon excitation of the peripheral pain fiber, the neuron, with the cell body residing in the DRG, is activated, and this may result in the upregulation of several marker genes, among which substance P, CGRP, NPY, galanin, NAV1.7, P2RX3, α2δ1, ATF3, and GAP43, depending on the cause and nature of the pain [13].

The hypothesis of this study is that S100A8/9 induces pain as by direct activation of afferent nerve endings and by sensitization of the peripheral nervous system locally in the DRG, where it is produced by infiltrating monocytes.

Synovitis is studied in streptococcal cell wall (SCW) arthritis, which is based on local activation of the synovium by intra-articular deposition of SCW. SCW bind to TLR2 rather than to TLR4 [14], which makes this a good candidate to study the involvement of S100A8/9, a known TLR4 ligand, in pain perception.

Several methods to measure different aspects of pain have been described [15]. In this study, we used gait analysis and the incapacitance tester to study pain upon static and dynamic loading and von Frey testing to determine allodynia, as a measure for pain sensitization.

## Materials and methods

### Animals

Synovial activation was elicited in male C57BL/6 mice and in *S100a9*^−/−^ mice backcrossed to the C57BL/6 background for more than 12 generations. Myeloid cells of *S100a9*^−/−^ mice also lack S100A8 at the protein level [16]. Mice were used between the age of 14 and 16 weeks. All animal studies were conducted according to the Dutch law and approved by the Dutch Central Animal Experimentation Committee (project 2015-0014). A total of 80 mice were used, 6–8 per experimental group.

### SCW arthritis induction

SCW were produced as described previously [17]. After randomization, unilateral arthritis was induced by intra-articular injection of 25 μg SCW (rhamnose content) in 6 μL PBS into the right knee joint of 20 WT mice and 10 *S100a9*^−/−^ mice. As a sham control, saline was injected into the right knee joint of 20 WT and 10 *S100a9*^−/−^ mice. At day 1 and day 7 after induction, serum was collected and S100A8/9 levels were determined using an ELISA that was designed in-house [18].

### ^99m^Tc uptake measurement

To quantify joint swelling as a measure for inflammation, the uptake of ^99m^Tc-pertechnetate was measured [19]. The signal was expressed as a ratio relative to the naive contralateral knee joint. Briefly, mice were injected subcutaneously with 12 μCi ^99m^Tc-pertechnetate and sedated. After 30 min, the amount of radioactivity was assessed by measuring the gamma radiation with the knee in a fixed position, using a collimated NaI scintillation crystal. Swelling was measured at baseline and at days 1 and 7 after injection.

### Isolation of the knee joints and DRG

On days 1 and 7 after induction of SCW arthritis, the knee joints were isolated and fixed in 4% formaldehyde, decalcified in formic acid, and subsequently dehydrated and embedded in paraffin. From a part of the murine joints, synovium was isolated for the preparation of washouts and RNA isolation. Briefly, the synovium was isolated from the murine knee joints using a standardized method comprising removal of the quadriceps and section of the patella ligament. Tissue was put in RPMI (Gibco, Invitrogen, Carlsbad, CA, USA), enriched with 0.1% BSA (Sigma–Aldrich) for 1 h at room temperature. Prior to incubation, two 3-mm biopsies were taken for RNA isolation. DRG were isolated at the L3–5 levels of the spinal column. Briefly, the spines were isolated and cut in half transversely, and the ipsilateral L3–5 were removed. DRG were fixed in 4% formaldehyde, subsequently dehydrated, and embedded in paraffin. Paraffin sections were cut at 7 μM or 5 μM and mounted on coated slides. Hematoxylin/eosin (H&E) staining was performed to study the inflammatory cells and tissue morphology. Of the knee joints, inflammation scored using an arbitrary scoring system. The severity was determined using an arbitrary score (0–3), performed blindly by two observer: 0, no influx; 1, mild cellularity; 2, higher cellularity; and 3, very high cellularity. Immunohistochemical (IHC) staining was performed on S100A8 and S100A9 in WT mice. As primary antibodies, anti S100A8 and S100A9 were used that were prepared as described previously [5]. On DRG, IHC staining was performed for F4/80, to detect monocytes/macrophages, and for NAV1.7 (Alomone Labs, asc-008). For all IHC detections, isotype-matched IgG was used as a negative control. For S100A8, S100A9, and NAV1.7, IHC staining was scored in a semi-quantitative manner by assessing the diaminobenzidine (DAB) intensity using ImageJ (NIH, Bethesda). Since DAB intensity is not linearly related to expression, the staining is qualified as semi-quantitative. Briefly, photomicrographs were corrected for background, area of interest was selected, and the mean intensity of the area was assessed using the “H DAB” plugin after color deconvolution. Quantification of F4/80 staining in the DRG was done by counting the amount of positive cells per field of view after deconvolution.

### Assessment of pain behavior

In order to reliably measure pain in animal models, using more than one way to measure pain behavior in experimental models is highly recommendable [15]. None of the available pain tests for rodents is specific only for pain, since in all cases, behavior is studied, and behavior can easily be influenced by other parameters. Therefore, in our experiments, extreme care was taken to include a proper adjustment period for acclimatization, sham controls were used, and baseline measurements were taken at 3 alternate days before the start of the experiments. By taking these precautions, and by combining several methods, pain measurements can be reliably performed, and several groups have shown that these methods reflect pain behavior since the use of analgesics normalizes behavior [20]. All measurements were performed by the same observers (AB, EBD, and EG) who were blinded for treatment and phenotype. Mice were randomized prior to the start of the experiments. The methods we used were (a) incapacitance testing, (b) gait analysis, and (c) von Frey testing.

#### Incapacitance tester

To assess the weight distribution between the hind paws, we used the Linton incapacitance tester (Linton Instruments, UK). Briefly, mice were put on the device and due to the dimensions of the device, mice were forced to stand on their rear legs, and the left and right legs were positioned on two separate scales. Due to the hyperactive nature of mice in general, using the incapacitance tester can be challenging, but reliable results have been obtained by many groups. We used a functionality of the incapacitance tester that only starts measuring when the mouse is in a stable position, as reflected by stable readings for at least 4 s, to ensure a reliable measurement. The measurement itself lasted 1 s. Per mouse, 6 serial measurements were made, and the mean was calculated. Before the start of the measurements, mice were acclimatized to the incapacitance tester 3 times before the start of the measurements at day − 7. Three baseline measurements were made on days − 7, − 3, and day 0. Subsequently, the effect of synovitis was measured at day 1 and day 7 after injection of SCW. The results were expressed as the percentage of total weight on the arthritic right leg compared to the combined weight on the right and unaffected left leg.

#### Gait analysis using Catwalk-XT

To measure changes in gait parameters induced by injection of SCW, and to assess whether differences were present between WT and S100A9^−/−^ mice, gait analysis was performed using the Catwalk-XT (Catwalk XT®, Wageningen, The Netherlands). Within this system, the mice are allowed to walk freely across a glass plate. A high-speed camera is used to capture suitable runs over a distance of 30 cm, which are selected using software settings: maximal variation in run speed, 60%; minimal time, 1 s; maximal time, 4 s. If these criteria were met, the run was considered valid. A full stop of the mouse during a run would render the run invalid. Per mouse, 3 valid runs were obtained, and for all data, the mean over these runs was calculated. Footprints and footfall patterns were identified and digitally analyzed. Changes in the footprint area, the duration of the stand phase of each paw, and the terminal dual stance were measured as a measure for pain behavior. The footprint area was calculated as the mean width × length for each paw. The stand phase was calculated as the mean duration of contact with the glass of each paw separately. The mean terminal dual stance represents the duration of the overlap of the stand phase between the left and right joint, at the moment the left paw is placed. It represents the time that both hind paws are loaded in the final stand phase of the right hind limb. An increase in time is a measure for increased pain, as this can be considered an attempt to decrease the loading of the right joint. This parameter quantifies what could be called “limping.”

#### Assessment of allodynia using von Frey hairs

Mechanical allodynia in mice was tested using the up–down staircase method as described by Dixon [21]. In short, mice were placed on a metal grid (with 3-mm-diameter holes) within small Plexiglas cubicles, and a set of 10 calibrated von Frey fibers (0.07, 0.16, 0.4, 0.6, 1, 1.4, 2, 4, 6, and 8 g, BioSeb, Vitrolles, France) was applied perpendicular to the plantar surface of the hind paw until the fibers bowed and then held for 2 s. Paw withdrawal was scored if within 5 s of removal of the fiber the paw was withdrawn or licked. Each fiber was applied 3 times, and a positive withdrawal response was scored if 2 or 3 out of 3 applications resulted in paw withdrawal. All testing and scoring were performed by one researcher. Testing was initiated with the 0.6-g fiber; if the response was negative, a fiber with increased force was applied, and in the event of a positive response, a weaker stimulus was applied. Sequential measurements were separated by at least 5 min. When a fiber resulted in a positive withdrawal response, 4 more additional fibers were tested. The 50% withdrawal threshold was interpolated as described in detail by Chaplan et al. [22].

### Isolation of RNA (synovium and DRG) and subsequent qPCR

Gene expression was determined using quantitative real-time PCR (qRT-PCR). DRG for RNA isolation were homogenized in RLT buffer using the MagNA Lyser Instrument (Roche). Total RNA was isolated using the RNeasy MinElute kit (Qiagen) with a Proteinase K step according to the manufacturer’s protocol. The RNA concentration was determined using a Nanodrop spectrophotometer and subsequently reverse transcribed into cDNA. qRT-PCR was performed using specific primers (Table 1) and the SYBR Green Master Mix in the Applied Biosystems StepOnePlus real-time PCR system (Applied Biosystems). Reactions were presented as minus delta threshold cycle Ct (−ΔCt) values, calculated by correcting the negative threshold cycle (−Ct) of the gene of interest to the −Ct of the reference gene GAPDH.

**Table 1:** Primers to measure expression in synovium and/or DRG using Q-PCR

Gene	Forward primer	Reverse primer
*α2δ1(A2d1)*	5′-gtcacactggattttctcgatgc-3′	5′-gggtttctgaatatctggcctga-3′
*Atf3*	5′-gaggattttgctaacctgacacc-3′	5′-ttgacggtaactgactccagc-3′
*Cfos*	5′-aaacccatcaccatcttcca-3′	5′-gtggttcacacccatcacaa-3′
*Cgrp*	5′-ttgtcagcatcttgctcctgtac-3′	5′-gcctgggctgctttcca-3′
*Galanin*	5′-ggcagcgttatcctgctagg-3′	5′-ctgttcagggtccaacctct-3′
*Gap43*	5′-tggtgtcaagccggaagataa-3′	5′-gctggtgcatcacccttct-3′
*Gapdh*	5′-ggcaaattcaacggcaca-3′	5′-gttagtggggtctcgctcctg-3′
*Il1b*	5′-ggacagaatatcaaccaacaagtgata-3′	5′-gtgtgccgtctttcattacacag-3′
*Il6*	5′-caagtcggaggcttaattacacatg-3′	5′-attgccattgcacaactcttttct-3′
*Kc*	5′-tggctgggattcacctcaa-3′	5′-gagtgtggctatgacttcggttt-3′
*Mcp1*	5′-ttggctcagccagatgca-3′	5′-cctactcattgggatcatcttgct-3′
*Nav1.7*	5′-cgacagcggcacaactaatc-3′	5′-agaatgcttgctctgctcatg-3′
*Ngf*	5′-tcgggccagtatagaaagct-3′	5′-ggggagcgcatcgagtttt-3′
*Npy*	5′-atgctaggtaacaagcgaatgg-3′	5′-tgtcgcagagcggagtagtat-3′
*P2rx3*	5′-aaagctggaccattgggatca-3′	5′-cgtgtcccgcacttggtag-3′
*S100a8*	5′-tgtcctcagtttgtgcagaatataaat-3′	5′-tttatcaccatcgcaaggaactc-3′
*S100a9*	5′-ggcaaaggctgtgggaagt-3′	5′-ccattgagtaagccattcccttta-3′
*SubstanceP (Tac1)*	5′-attcctttgttggactaatgggc-3′	5′-acgtcttctttcgtagttctgc-3′
*Tnfa*	5′-cagaccctcacactcagatcatct-3′	5′-cctccacttggtggtttgcta-3′
*Trka*	5′-gcctaaccatcgtgaagagtg-3′	5′-ccaacgcattggaggacagat-3′

### Statistical analysis

Statistical analyses were performed using Graphpad Prism version 5.03. Differences between the groups were tested using a one-way or two-way analysis of variance (ANOVA). Differences in histology were tested with a non-parametric Mann–Whitney *U* test. *p* values lower than 0.05 were considered significant. Results are expressed as mean values ± standard deviation (SD).

## Results

### Serum levels and synovial expression and release of S100A8 and S100A9 are increased upon SCW injection

**Fig. 1:**
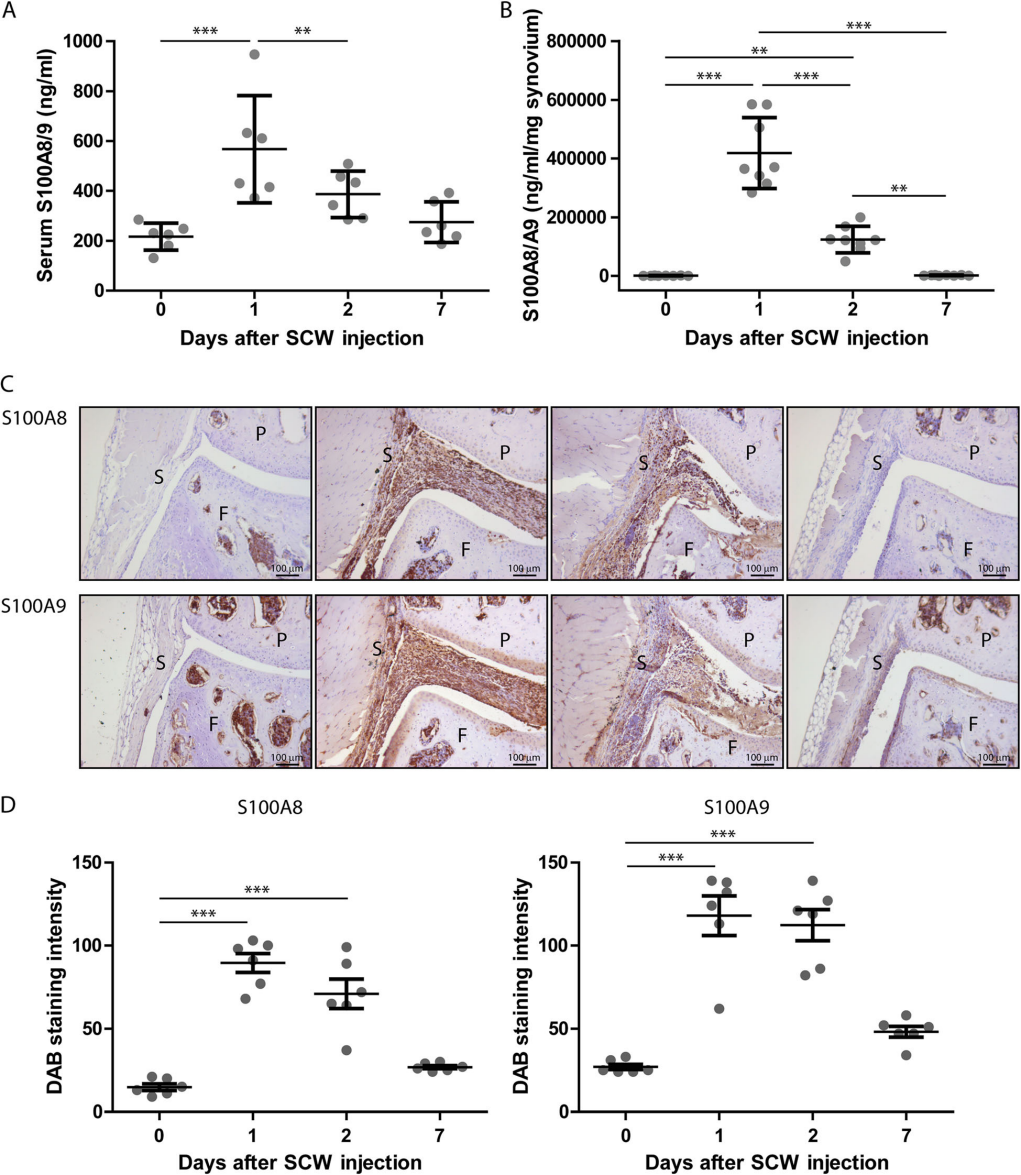
S100A8 and S100A9 expression is increased during SCW arthritis. **a** In synovial washouts, S100A8/9 was detected using an ELISA. Washouts were obtained by washing synovial explants for 2 h in the culture medium at RT. Explants were isolated at baseline and at days 1, 2, and 7 (**b**). Using immunohistochemistry, S100A8 and S100A9 (**c**) were detected in the knee joints at baseline and at days 1, 2, and 7 after induction of synovitis. S100A8/9 is clearly increased at days 1 and 2 and wanes thereafter (*n* = 6 for all groups). **d** This is quantified using ImageJ. One-way ANOVA with Tukey multiple comparison test was performed. ***p < 0.001; **p < 0.01; *p < 0.05. P, patella; F, femur; S, synovium

First, we determined whether in the acute synovitis model of SCW induced arthritis in C57Bl/6 mice, S100A8 or S100A9 genes and proteins were expressed. After a single i.a. SCW injection, the expression of both *S100a8* and *S100a9* mRNA was significantly upregulated locally in the synovium. The peak expression was found on day 1 (resp. 29- (ddCt 5.4 ± 2.1) and 34-fold increase (ddCt 5.8 ± 1.8) compared to sham), and levels decreased at day 2- to 8-fold increase for both (ddCt resp. 3.0 ± 1.6 and 3.0 ± 1.2) compared to sham and were back to baseline at day 7 (ddCt resp. 0.8 ± 1.1 and 0.9 ± 1.5). This upregulation in *S100a8* and *S100a9* mRNA was reflected by increased protein levels in serum and synovial washouts (Fig. 1a, b).

Using immunohistochemistry we demonstrated that levels of both S100A8 and –A9 peaked at day 1, decreased slightly at day 2 and were low, but still above baseline at day 7 after SCW injection (Fig. 1c, d).

### Increased S100A8/A9 expression after SCW injection does not contribute to the development of inflammation

**Fig. 2:**
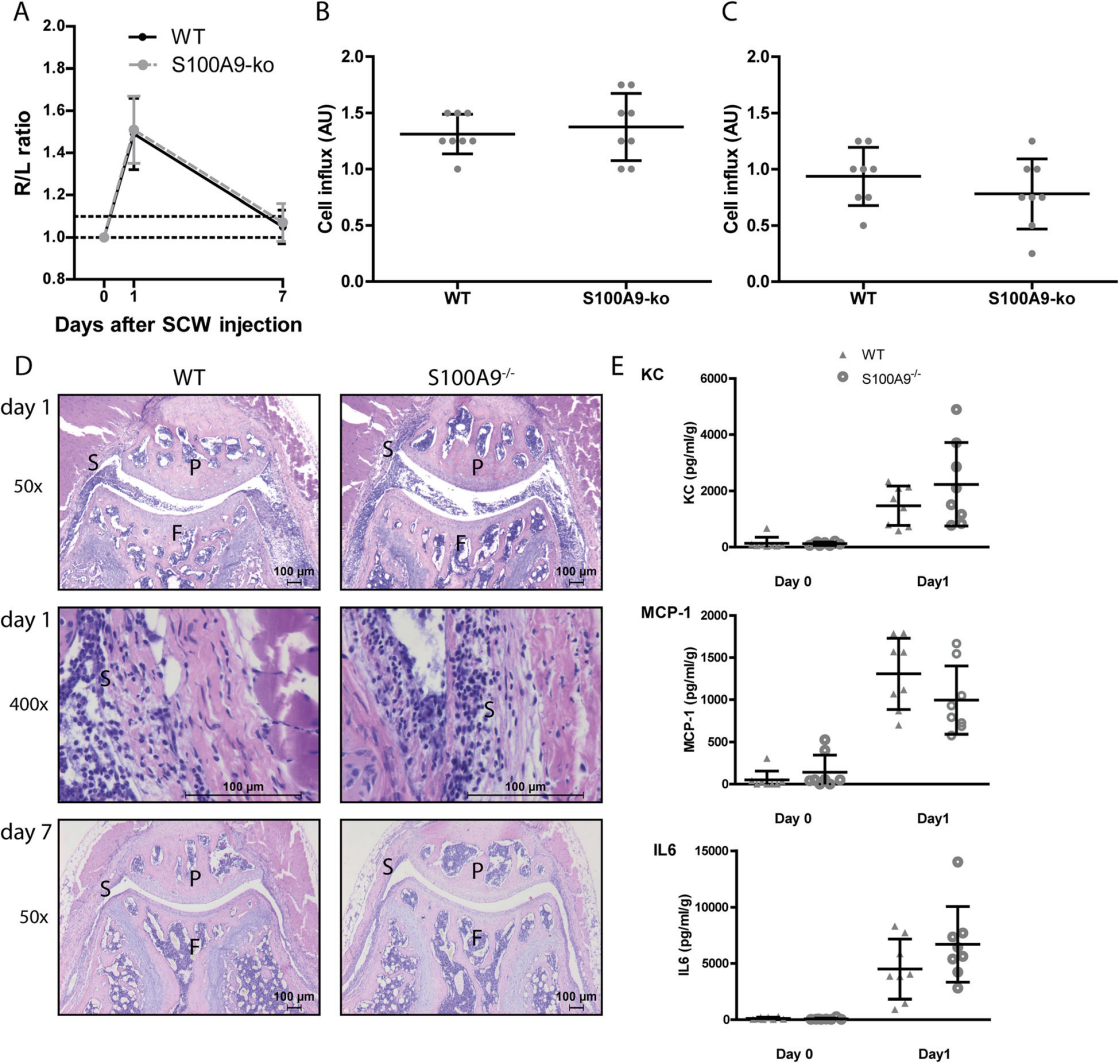
Synovitis is not different in WT versus *S100A9*^*−/−*^ mice. **a** Swelling was the same for both WT and *S100A9*^*−/−*^ mice and was high at day 1 and back to baseline at day 7 (below threshold ratio of 1.1). Cell influx showed no difference between WT mice and *S100A9*^*−/−*^, both at days 1 (**b**, **d**) and 7 (**c**, **d**) after the induction of SCW arthritis. Protein levels of MCP-1, KC, and IL-6 were increased but not different between WT and *S100a9*^*−/−*^ mice (**e**) (*N* = 16 for **a**; *n* = 8 for other panels). Student *t* test was used for **a**. Two-way ANOVA was used with a Tukey post hoc test for the other panels. P, patella; F, femur; S, synovium

To study the involvement of S100A8/9 in synovitis, we first determined swelling of the knee joint at day 1 and at day 7 after SCW injection. Swelling increased, as determined by measuring the ^99m^Tc-pertechnetate uptake R/L ratio, 1 day after injection of SCW in WT mice (Fig. 2a). At day 7, joint swelling was down to baseline levels. In *S100a9*^−/−^ mice, effectively lacking both S100A9 and S100A8, swelling was induced to comparable levels as in WT mice. Swelling at day 7 was not different from baseline levels. To determine whether S100A8/9 deficiency leads to changes in cell influx, we scored local inflammation using histology (Fig. 2b–d). Inflammation was high at day 1 and was decreased but still present at day 7 after induction. Interestingly, however, no differences in cell numbers and cell types could be observed between WT and *S100a9*^−/−^ mice (Fig. 2d). Mediators for inflammation, except TNFα and IL-1, were increased in synovial washouts during SCW arthritis, but none of the inflammatory mediators (KC, MCP-1, IL-6) were significantly different between WT and *S100a9*^−/−^ (Fig. 2e).

### SCW injection leads to increased nociceptive pain behavior in the affected leg, which is absent in S100a9^−/−^ mice

**Fig. 3:**
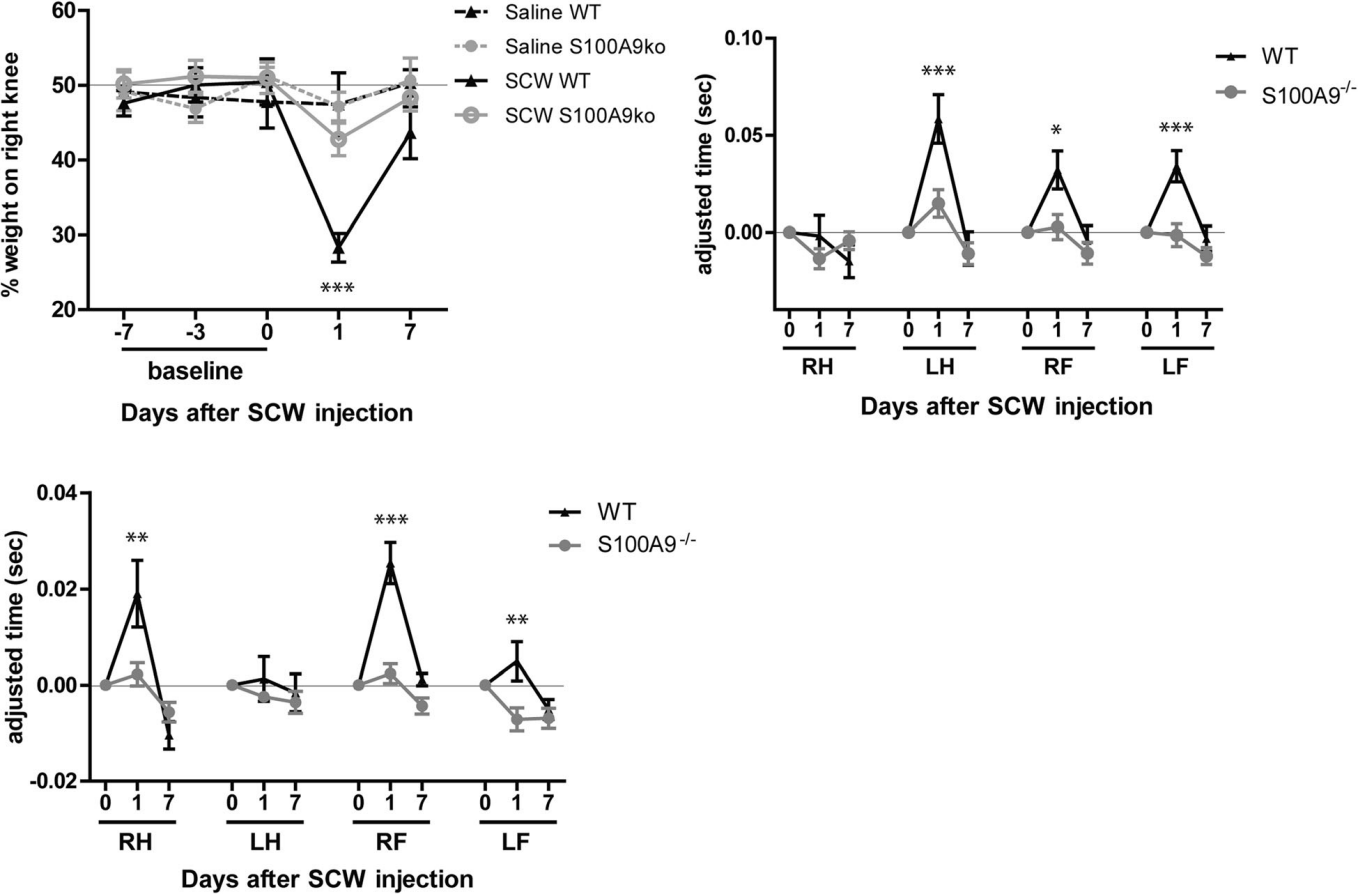
*S100A9*^*−/−*^ mice show less pain behavior than WT mice in SCW synovitis. **a** Wt mice showed a dip in the loading of the right, affected limb, while *S100a9*^−/−^ mice did not. Although the stand phase of WT mice was not different in the affected right hind limbs from controls, all other limbs showed an increased stance (**b**). Particularly at the end of each stand of the affected joints, the left limb was used to unload the right hind limb, as reflected in the TDS (**c**) in WT but not *S100A9*^−/−^ mice. Found in two separate experiments (*n* = 8 each). Two-way ANOVA with a Tukey post hoc test (*p < 0.05; **p < 0.01; ***p < 0.001)

The lack of effect of S100A8/9 on inflammation presented us with the perfect conditions to determine the role of S100A8/9 in pain irrespective of inflammation. We first measured pain behavior defined by static loading of the right and left paws using an incapacitance tester. At day 1 after induction, the percentage of total weight on the right hind leg decreased from approximately 50% at baseline to just below 30%, *p* < .001 (Fig. 3a). In contrast, mice lacking S100A9 showed no shift. Seven days after induction, incapacitance normalized almost completely to 44% in the WT and 48% in the *S100a9*^−/−^ and was no longer significantly different from saline-injected controls.

Next, animals were allowed to walk freely, and gait was monitored at day 1 after injection for 2 parameters, the stand phase of each paw, and the terminal dual stance (TDS). Remarkably, the stand phase of the right hind leg was unchanged 1 day after SCW injection, both in WT and *S100a9*^−/−^ mice. However, in WT mice, the stand phase in the remaining legs, left hind and right and left front legs, increased significantly (respectively *p* < .001, *p* < .05, and *p* < .001). This increase was not observed in *S100a9*^−/−^ mice (Fig. 3b). Next, the TDS was determined from the Catwalk data. This parameter can be described as “limping.” The TDS significantly increased in wild-type mice after injection of SCW, resulting in “limping” behavior (Fig. 3c). In contrast, no change in TDS was observed in the *S100a9*^−/−^ mice with SCW arthritis, underlining the role of S100A8/9 in pain perception.

### SCW-induced mechanical allodynia does not differ between WT and S100A9^−/−^ mice

**Fig. 4:**
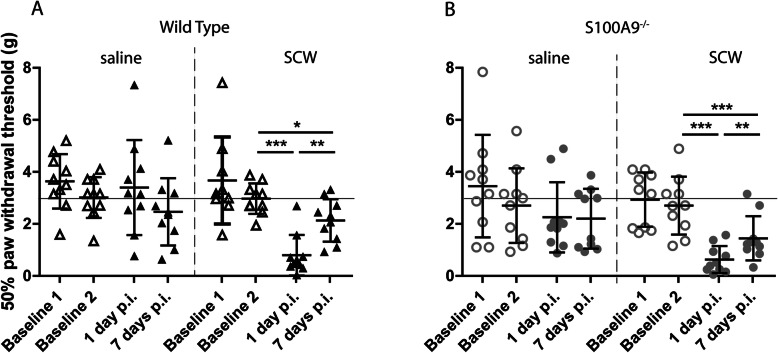
Allodynia is induced during SCW synovitis, but not different between WT and *S100A9*^*−/−*^. Injection of saline did not induce changes in the pain threshold, but injection of SCW lowered the threshold significantly at day 1 (**a**). Although the threshold recovered partly at day 7, it was still significantly different from baseline. Allodynia in *S100a9*^−/−^ mice did not differ from WT and showed a highly similar pattern, indicating that S100A8/9 is not involved in sensitization. Significance was tested using a two-way ANOVA at *n* = 8 per group. A Tukey post hoc test was performed to identify significantly different means (*p < 0.05; **p < 0.01; ***p < 0.001)

To determine whether S100A8/9 are contributing to pain sensitization, we measured hind paw mechanical allodynia using von Frey filaments. At day 1 after SCW injection, allodynia could be clearly demonstrated (*p* < .001) in WT mice (Fig. 4a). Saline-injected animals did not differ from baseline and had a 50% paw withdrawal threshold of approximately 3 g, whereas SCW-injected mice showed a dip of approximately 1 g at day 1 after injection, which returned to baseline levels at day 7. In contrast to the parameters for general pain perception, allodynia in *S100a9*^−/−^ was present (*p* < .001) and did not differ from WT (Fig. 4b).

### SCW synovitis does not cause cell influx or pro-inflammatory response in the DRG and no differences between WT and S100A9^−/−^ mice

**Fig. 5:**
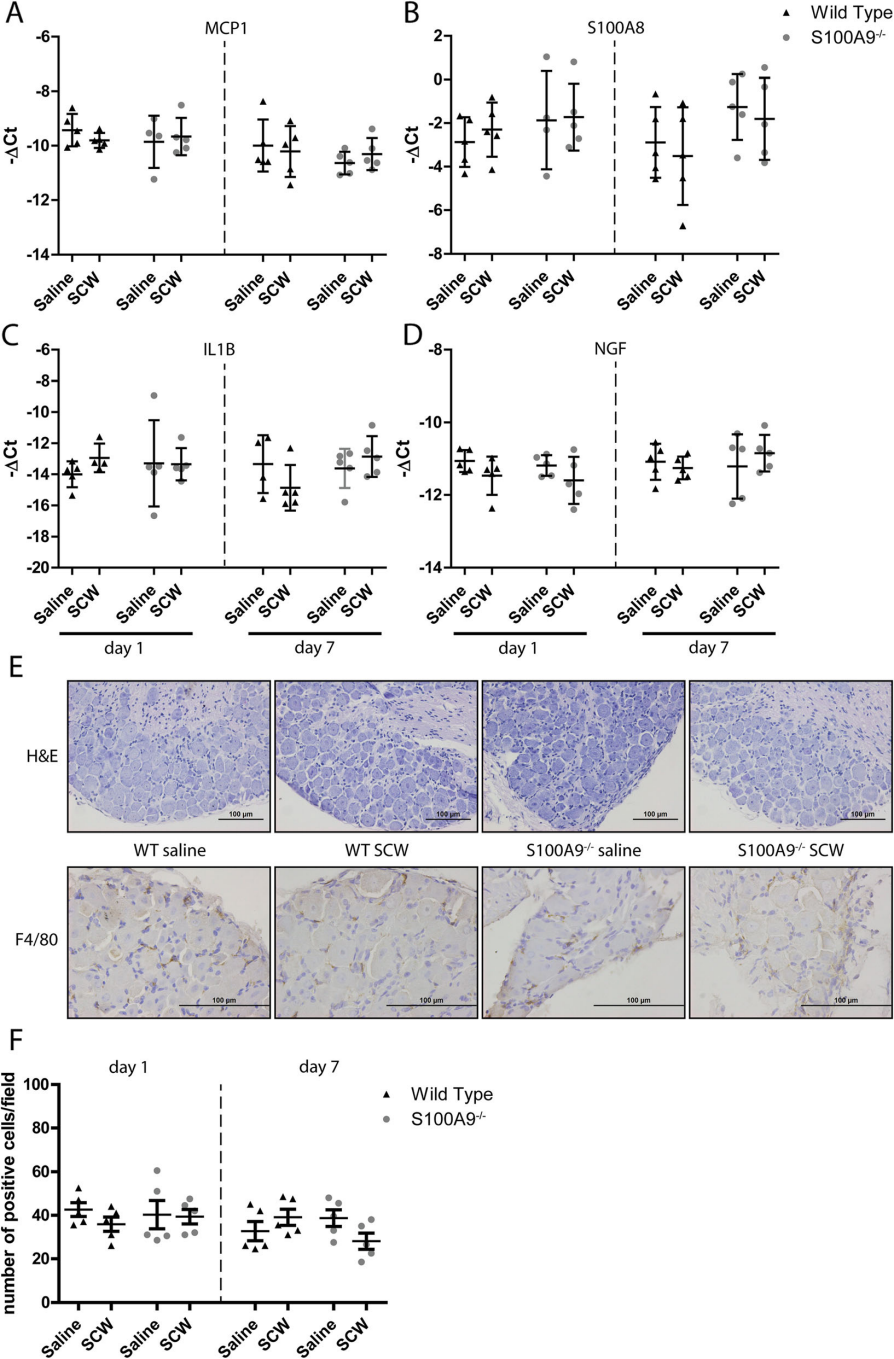
No signs for inflammatory reaction in the DRG during acute synovitis. Ipsilateral DRG of both WT and *S100a9*^−/−^ mice were isolated and used for both RT-PCR of several genes involved in inflammation (*n* = 5) and for histological examination (*n* = 5). No significant differences in the expression of inflammation-related genes (MCP1 (**a**), S100A8 (**b**), IL-1B (**c**), and NGF (**d**)) were observed. When the tissue was examined histologically, the H&E staining (magnification × 200) did not show any alteration in mice injected with SCW from mice injected with saline. Also, no differences between WT and *S100a9*^−/−^ mice were observed (**e**). When monocytes/macrophages were specifically detected using F4/80 (magnification × 400), after quantification, no differences were observed between all groups (**e**, **f**)

To study the possible mechanisms that underlie differences in pain perception between WT and *S100a9*^−/−^, we determined whether the inflammatory response in the relevant DRGs (L3-L5) was different between WT and *S100a9*^−/−^. In the joint, S100A8/9 in WT may bind to the afferent neuronal ending, thus causing activation of the neuronal bodies in the DRG. We did not find increased expression of MCP-1 in the DRG 1 or 7 days after induction of synovitis (Fig. 5a). Also, other mediators that may signify cell influx or inflammation (S100A8, IL-1β, NGF, and TNFα, the latter not shown) were unchanged (Fig. 5b–d). Histological examination of the DRG proved a lack of cell influx upon induction of synovitis. This was confirmed by immunohistochemical detection of F4/80, which was not different in DRG of SCW versus saline-injected mice (Fig. 5e, f).

### SCW injection results in increased expression of neuron activation markers in DRG of WT mice but not in S100a9^−/−^ mice

**Fig. 6:**
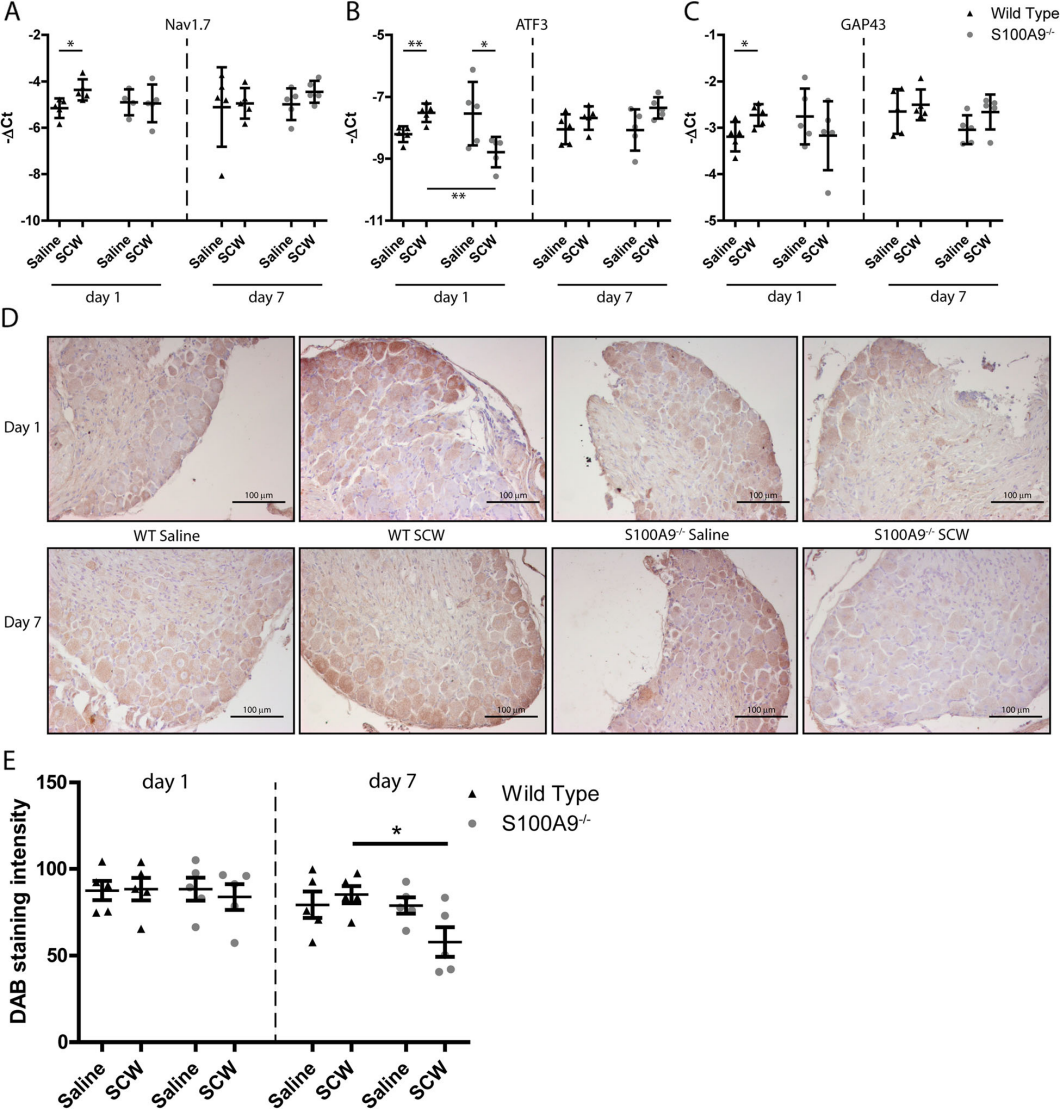
Of a panel of activation markers, ATF3, NAV1.7, and GAP43 are differentially expressed in DRG of arthritis WT versus *S100A9*^*−/−*^ mice. Three neuronal activation markers showed a significant increase in WT mice after SCW injection: NAV1.7 (**a**), ATF3 (**b**), and GAP43(**c**). These differences were found only 1 day after the injection of SCW. In *S100a9*^*−/−*^ mice, no increase in these markers were found, for ATF3 even a small decrease. At day 7, protein level NAV1.7 also seemed lower in *S100a9*^*−/−*^ compared to saline-injected mice, whereas in WT mice, the expression seemed increased (**d**; magnification × 200). This was quantified, and protein expression was lower at day 7 in S100a9^−/−^ mice, compared to WT (**e**). Significance was tested using a two-way ANOVA at *n* = 5 per group. A Tukey post hoc test was performed to identify significantly different means (*p < 0.05; **p < 0.01; ***p < 0.001)

Now that we could not demonstrate regulation of inflammatory response genes in the DRG, we detected genes that are involved in neuron function in the DRG and may serve as markers for activation or damage: Substance P, CGRP, NPY, galanin, NAV1.7, P2RX3, α2δ1, ATF3, and GAP43. First, we determined levels of mRNA expression of these genes in WT mice injected i.a. with SCW and compared this to WT mice that received saline (Table 2). One day after induction of SCW arthritis, 3 genes of this panel were upregulated: NAV1.7 (1.7-fold), ATF3 (1.8-fold), and GAP43 (1.9-fold) (Fig. 6a–c). Subsequently, regulation of these genes in DRG of *S100a9*^−/−^ mice after induction of SCW arthritis was absent. In contrast, even a downregulation of ATF3 compared to WT mice was observed (2.1-fold). At day 7 after induction of SCW, none of the genes were differentially expressed, in WT nor in *S100a9*^−/−^. The mRNA findings of NAV1.7 were confirmed by immunohistochemistry on DRG, where enhanced staining was found in WT compared to *S100a9*^−/−^, although protein levels were not significantly different between WT and *S100a9*^−/−^ until day 7 (Fig. 6d, e).

**Table 2:** Q-PCR results DRG. dCT ± SD *p < .05. Significance was calculated compared to the saline

	Day 1				Day 7			
	WT		***S100A9***^**−/−**^		WT		***S100A9***^**−/−**^	
	Saline	SCW	Saline	SCW	Saline	SCW	Saline	SCW
*α2δ1(A2d1)*	− 13.1 ± 0.7	− 12.4 ± 0.9	− 12.5 ± 1.9	− 13.1 ± 1.2	− 12.8 ± 1.2	− 12.2 ± 0.5	− 13.1 ± 0.6	− 13.8 ± 1.7
*Atf3*	− 8.2 ± 0.3	− 7.5 ± 0.3^*^	− 7.5 ± 0.9	− 8.8 ± 0.5^*^	− 8.0 ± 0.5	− 7.7 ± 0.4	− 8.1 ± 0.7	− 7.4 ± 0.3
*Cfos*	− 10.1 ± 0.5	− 10.9 ± 0.4	− 10.6 ± 0.4	− 10.6 ± 0.4	− 11.2 ± 0.5	− 11.0 ± 0.4	− 9.8 ± 1.6	− 9.5 ± 1.3
*Cgrp*	− 0.4 ± 0.4	− 0.7 ± 0.6	− 0.7 ± 0.4	− 0.3 ± 0.5	− 0.7 ± 0.3	− 0.5 ± 0.3	− 0.6 ± 0.2	− 0.5 ± 0.3
*Galanin*	− 5.8 ± 0.3	− 5.8 ± 0.4	− 5.9 ± 0.5	− 6.0 ± 1.4	− 5.6 ± 0.7	− 5.6 ± 0.6	− 5.7 ± 0.4	− 5.0 ± 0.5
*Gap43*	− 3.2 ± 0.3	− 2.7 ± 0.2^*^	− 2.8 ± 0.6	− 3.2 ± 0.7	− 2.6 ± 0.5	− 2.5 ± 0.3	− 3.0 ± 0.3	− 2.7 ± 0.4
*Nav1.7*	− 5.2 ± 0.4	− 4.4 ± 0.5^*^	− 4.9 ± 0.6	− 4.9 ± 0.8	− 5.1 ± 1.7	− 4.9 ± 0.7	− 5.0 ± 0.7	− 4.4 ± 0.5
*Ngf*	− 11.1 ± 0.3	− 11.5 ± 0.5	− 11.2 ± 0.3	− 11.6 ± 0.7	− 11.1 ± 0.5	− 11.3 ± 0.3	− 11.2 ± 0.9	− 10.8 ± 0.5
*Npy*	− 10.7 ± 0.3	− 11.4 ± 1.3	− 10.8 ± 1.1	− 11.7 ± 1.4	− 8.7 ± 1.1	− 8.5 ± 0.6	− 8.9 ± 0.5	− 8.5 ± 0.5
*P2rx3*	− 6.9 ± 0.4	− 6.8 ± 0.8	− 6.8 ± 0.6	− 7.0 ± 0.7	− 6.9 ± 0.4	− 6.1 ± 0.5	− 6.7 ± 0.6	− 6.6 ± 0.8
*Subst P*	− 1.6 ± 0.4	− 1.5 ± 0.6	− 1.8 ± 0.6	− 1.3 ± 0.3	− 1.6 ± 0.4	− 1.8 ± 0.2	− 1.8 ± 0.2	− 1.6 ± 0.3
*Trka*	− 8.5 ± 0.7	− 8.1 ± 1.1	− 8.2 ± 1.0	− 8.3 ± 0.5	− 8.5 ± 0.9	− 7.8 ± 0.5	− 8.7 ± 1.1	− 8.4 ± 0.4

## Discussion

Synovitis and bone marrow lesions have been proposed to determine OA pain [23]. In RA, it is well accepted that synovitis is an important source of pain, but also here, mechanisms are poorly understood [24]. An intriguing question is whether key mediators can be identified in the joint that are particularly involved in the generation and maintenance of pain caused by synovitis. S100A8/A9 is a potent mediator of synovitis and joint destruction during mouse and human OA and these alarmins are produced within the joint throughout the course of the disease. In the present study, we set out to determine if and to what extent the S100A8/9 heterodimer is involved in pain that is mediated by synovitis. We clearly demonstrate that the heterodimer S100A8/9 is indeed involved in pain behavior in an experimental model for synovitis. S100A8/9 seem particularly important in nociception, since we found a clear normalization of joint loading and gait in *S100a9*^−/−^.

SCW synovitis is based on the activation of TLR2, rather than TLR4, which would disturb the measurement of isolated effects of the TLR4 ligand S100A8/9 [14]. Interestingly, in contrast to other models [6, 7, 25], the inflammation in the SCW model was not mediated by S100A8/A9. This allowed us to study the direct effect of S100A8/A9 in pain, independent of the differences in inflammation. The SCW arthritis model is a generalized model for acute synovitis. The infiltrate is characterized by cells of the innate immune system, like monocytes, macrophages, and PMN, rather than lymphocytes, cell types relevant for both RA and OA*.* Inflammation is an important source of pain in many processes, including arthritic diseases [10, 26]. It has been recently shown that a 32-mer aggrecan fragment is a potent activator of TLR2 and is involved in inflammation and pain in models for OA [27, 28].

Much is still unknown about the involvement of the DAMP S100A8/9, in pain perception. We studied S100A8/9 involvement in 2 different aspects of pain: (1) acute inflammatory or nociceptive pain, caused by direct excitation by S100A8/9 of the relevant afferent nerve fibers, likely via TLR4 or RAGE, directly resulting in pain [8, 29]; (2) sensitization, induced by binding of TLR4 or RAGE, and resulting in increased excitability of the afferent nerve fiber, via cell influx into the DRG or via regulation of nociceptors or ion channels.

By comparing the differential effects of S100A8/9 on the specific parameters, we deducted the involvement of S100A8/9 in these different aspects of pain. The first indication that pain caused by synovitis is mediated by S100A8/A9 came from our finding that static weight bearing, determined with the incapacitance tester [30, 31], was unchanged in *S100a9*^−/−^ but not in WT mice. Gait was analyzed to measure the dynamic loading of the paws and “limping” [32]. These parameters were quantified as (1) the mean stand phase of all four paws and (2) the TDS. Both parameters have been described before to reflect pain behavior [33, 34]. We found an increase in stand phase in the unaffected paws in WT mice, rather than a decrease of the stand phase of the arthritic paw. It is assumed that the loading of unaffected limbs is increased to spare the affected limb. This effect has been described before in a rat model for arthritis [35]. TDS of the right hind paw is considered an indicator of pain, and changes represent “limping” behavior. This clear demonstration of lack of pain behavior when S100A8/9 is absent affirms the importance of S100A8/9 in general pain behavior during synovitis.

To study the second aspect of pain, sensitization, we determined the pressure-pain threshold (tactile allodynia) using von Frey filaments [36, 37]. Although clearly present in WT and *S100A9*^−/−^ mice, allodynia was equal between the two groups, excluding a role for S100A8/9 in sensitization in acute synovitis.

Together, these data strongly suggest a role for the TLR4 ligand S100A8/9 in acute nociception rather than modulation of pain sensitivity. This is in line with a recent finding, in which TLR4 deficiency did not alter sensitization [10]. Although S100A8 stimulation caused excitation of afferent neurons, when a model for OA, destabilization of the medial meniscus (DMM), was induced in the knee joints of *Tlr4*^−/−^ mice, no effect on sensitization was demonstrated. In contrast to these findings, a study in which LPS was injected in the paw of WT and *Tlr4*^−/−^ mice did demonstrate a role for TLR4 in this process [38]. This indicates that endogenous TLR4 ligands as formed in (osteo) arthritis models may play a redundant role. S100A8/9 can bind to RAGE, but most literature suggests a role for RAGE in neuropathic pain and central sensitization, for which the current model is not suitable [39]. We therefore cannot claim a role for this receptor. Future experiments could include i.a. injection of S100A8 or S100A9 homodimers to test the hypothesis that these compounds induce an acute pain response, and using specific knockout mice, this could shed light on the receptors that are involved in this. However, it would be difficult to determine whether this would be a direct effect on pain behavior or indirect through the mediation of inflammation.

To shed more light on the cellular and molecular processes that may underlie the differences in pain behavior, we studied the DRG, where the cell bodies of the Aδ- and C-fibers reside [40]. Ipsilateral DRG from L3–L5 were pooled, since the neuronal bodies of the afferents from the knee joint reside at these levels, using retrograde fluorogold staining [41]. We tested the expression of markers for neuronal inflammation, activation, and neuropathy. No induction of markers for inflammation was found, which is in contrast to previous studies which were performed in a model for OA, the DMM [10, 42]. Also in contrast to these studies, we did not observe an influx of monocytes in DRG, likely explained by the lack of upregulation of MCP-1 by the DRG. The lack of DRG inflammation in synovitis during acute SCW-induced arthritis refutes our hypothesis, derived from the DMM-data, that influx of cells or production of MCP-1 by the DRG would be responsible for the sensitization in both WT and *S100a9*^−/−^ mice [10, 43, 44]. An obvious difference between both models is the acute nature of the SCW arthritis compared to the DMM, with long-lasting mild synovitis. Whether the DRG inflammation during DMM is S100A8/A9-dependent is currently under investigation. Possibly in the chronic variant of the SCW model, DRG inflammation is comparable to DMM, which will be subject to future experiments [45]. The relevance of an acute flare of inflammation during OA for joint pathology was demonstrated elegantly in a study in rats where an acute inflammation was induced during an induced instability model for OA by intra-articular carrageenan injection. This indeed caused acute pain and also led to more pronounced end-stage pathology [46].

Stimulation of TLR4 by LPS leads to the expression of IL-1 and TNFα in the DRG [47]. In addition, TLR4 may play a role in the conversion of acute to chronic pain, which was demonstrated in a model for chronic arthritis, where TLR4 was involved in sustaining sensitization after the inflammation waned [48, 49].

Cytokines that are produced locally in the joint during arthritis, like IL-1, TNFα, IL-6, and IL-17, have been shown to modulate neurons [50]. However, in osteoarthritis, cytokine levels are considerably lower and therefore may have less impact. In contrast, levels of S100A8/9 are high [6], and therefore, S100A8/9 may play a particularly relevant role in OA pain. In vitro stimulation of DRG with S100A8 resulted both in the excitation of the neurons and the production of inflammatory mediators [10]. In a study in rats, macrophages were stimulated with LPS in co-culture with DRG neurons, and this led to apoptosis of neurons, suggesting a role for TLR4 in neuropathy [51].

Male mice showed TLR4-mediated hyperalgesia when challenged with LPS, whereas the role of TLR4 in allodynia was comparable between male and female mice [52]. This, and other studies, indicate sex as an important factor in pain. In the present study, experiments were performed in males only, which is a limitation of our approach.

The neuronal markers that were differentially expressed in the DRG were NAV1.7, ATF3, and GAP43, which were increased in WT DRG and not *S100a9*^−/−^. These markers appear related to both nerve injury and inflammatory pain since they are also expressed in collagen antibody-induced arthritis and signify activation of the Aδ- and C-afferents [13]. The fact that they are differentially expressed in *S100a9*^−/−^ is in line with the demonstrated difference in pain behavior. They are related to nerve injury and this suggests that local joint inflammation during SCW causes damage to peripheral afferent fibers [53]. However, we did not find clear signs of nerve injury at this early time point, like prolonged changes in pain behavior in this acute synovitis. Unfortunately, due to the experimental setup, we were not able to further study local processes. Possibly, the neuronal markers for tissue inflammation, CGRP, substance P, and α2δ1, but also TrkA, would have been differentially expressed [51, 54]. In a study in which the effect of local macrophage activation in the DRG was studied, activated macrophages induced CGRP production by afferent neurons. Our lack of monocyte influx may at least explain the lack of CGRP regulation [51]. Nevertheless, we were able to confirm the effects of S100A8/9 on pain behavior on a molecular level.

## Conclusions

Here, we clearly demonstrate a role for S100A8/9 in pain perception in a model involving synovitis. We did not find evidence for a role of S100A8/9 in sensitization. These findings have important implications for the development of pain treatment in both arthritis and OA. In both types of diseases, new treatments are much sought after. For OA, no effective long-term pain treatment is available, and these findings may provide evidence for a new approach, especially for inflammatory OA. TLR4 is expressed on primary sensory neurons and is implicated as a potential target in the treatment of pain during arthritis and OA pain in particular. The role of TLR4 in inflammation has been well established; however, targeted treatment or prevention of neuropathic pain with TLR4 antagonists is still under investigation. Increasing evidence suggests that the immune system plays an integral role in the transition to pain but no treatment is currently available to target this pathway [9]. Given the involvement of S100A8/9 in synovitis in several models [6, 7] and the direct role in pain that is demonstrated here, blocking S100A8/A9 may prove a successful strategy for pain management in arthritic diseases.

## Data Availability

Data and materials will be available upon request. Since no large databases are formed within these experiments, the data will not be deposited in a repository.

## Abbreviations

α2δ1: Voltage-dependent calcium channel subunit alpha-2/delta-1
ANOVA: Analysis of variance
ATF3: Activating transcription factor 3
CGRP: Calcitonin gene-related peptide
DAMP: Damage-associated molecular patterns
DMM: Destabilization of the medial meniscus
DRG: Dorsal root ganglion
ELISA: Enzyme-linked immunosorbent assay
GAP43: Growth-associated protein 43
H&E: Hematoxylin and eosin
IgG: Immunoglobulin G
IHC: Immunohistochemical
IL-1: Interleukin 1
KC: Keratinocyte chemoattractant
LPS: Lipopolysaccharide
MCP-1: Monocyte chemoattractant protein-1
NAV1.7: Sodium (Na) ion channel 1.7
NGF: Nerve growth factor
NPY: Neuropeptide Y
OA: Osteoarthritis
P2RX3: P2X purinoceptor 3
PMN: Polymorphonuclear neutrophils
qRT-PCR: Quantitative reverse transcription polymerase chain reaction
RA: Rheumatoid arthritis
RAGE: Receptor for advanced glycation end-products
SCW: Streptococcal cell wall
TDS: Terminal dual stance
TLR2: Toll-like receptor 2
TLR4: Toll-like receptor 4
TNFα: Tumor necrosis factor alpha
WT: Wild-type

## References

[1] Vos T, Barber RM, Bell B, Bertozzi-Villa A, Biryukov S, Bolliger (2015). Global, regional, and national incidence, prevalence, and years lived with disability for 301 acute and chronic diseases and injuries in 188 countries, 1990–2013: a systematic analysis for the Global Burden of Disease Study 2013. Lancet..

[2] Syx D, Tran PB, Miller RE, Malfait AM (2018). Peripheral mechanisms contributing to osteoarthritis pain. Curr Rheumatol Rep.

[3] Schaible HG (2018). Osteoarthritis pain. Recent advances and controversies. Curr Opin Support Palliat Care.

[4] Neogi T, Guermazi A, Roemer F, Nevitt MC, Scholz J, Arendt-Nielsen L (2016). Association of joint inflammation with pain sensitization in knee osteoarthritis: the multicenter osteoarthritis study. Arthritis Rheumatol..

[5] Vogl T, Stratis A, Wixler V, Völler T, Thurainayagam S, Jorch SK (2018). Autoinhibitory regulation of S100A8/S100A9 alarmin activity locally restricts sterile inflammation. J Clin Invest.

[6] van Lent PL, Blom AB, Schelbergen RF, Slöetjes A, Lafeber FP, Lems WF (2012). Active involvement of alarmins S100A8 and S100A9 in the regulation of synovial activation and joint destruction during mouse and human osteoarthritis. Arthritis Rheum.

[7] van Lent PL, Grevers L, Blom AB, Sloetjes A, Mort JS, Vogl T (2008). Myeloid-related proteins S100A8/S100A9 regulate joint inflammation and cartilage destruction during antigen-induced arthritis. Ann Rheum Dis.

[8] Wadachi R, Hargreaves KM (2006). Trigeminal nociceptors express TLR-4 and CD14: a mechanism for pain due to infection. J Dent Res.

[9] Bruno K, Woller SA, Miller YI, Yaksh TL, Wallace M, Beaton G (2018). Targeting toll-like receptor-4 (TLR4)-an emerging therapeutic target for persistent pain states. Pain..

[10] Miller RE, Belmadani A, Ishihara S, Tran PB, Ren D, Miller RJ (2015). Damage-associated molecular patterns generated in osteoarthritis directly excite murine nociceptive neurons through Toll-like receptor 4. Arthritis Rheumatol..

[11] Schaible HG (2012). Mechanisms of chronic pain in osteoarthritis. Curr Rheumatol Rep.

[12] Thakur M, Dickenson AH, Baron R (2014). Osteoarthritis pain: nociceptive or neuropathic?. Nat Rev Rheumatol.

[13] Su J, Gao T, Shi T, Xiang Q, Xu X, Wiesenfeld-Hallin Z (2015). Phenotypic changes in dorsal root ganglion and spinal cord in the collagen antibody-induced arthritis mouse model. J Comp Neurol.

[14] Abdollahi-Roodsaz S, Joosten LA, Helsen MM, Walgreen B, van Lent PL, van den Bersselaar LA (2008). Shift from toll-like receptor 2 (TLR-2) toward TLR-4 dependency in the erosive stage of chronic streptococcal cell wall arthritis coincident with TLR-4-mediated interleukin-17 production. Arthritis Rheum.

[15] Malfait AM, Little CB, McDougall JJ (2013). A commentary on modelling osteoarthritis pain in small animals. Osteoarthr Cartil.

[16] Manitz MP, Horst B, Seeliger S, Strey A, Skryabin BV, Gunzer M (2003). Loss of S100A9 (MRP14) results in reduced interleukin-8-induced CD11b surface expression, a polarized microfilament system, and diminished responsiveness to chemoattractants in vitro. Mol Cell Biol.

[17] van den Broek MF, van den Berg WB, van de Putte LB, Severijnen AJ (1988). Streptococcal cell wall-induced arthritis and flare-up reaction in mice induced by homologous or heterologous cell walls. Am J Pathol.

[18] Vogl T, Tenbrock K, Ludwig S, Leukert N, Ehrhardt C, van Zoelen MA (2007). Mrp8 and Mrp14 are endogenous activators of Toll-like receptor 4, promoting lethal, endotoxin-induced shock. Nat Med.

[19] Kruijsen MW, van den Berg WB, van de Putte LB, van den Broek WJ (1981). Detection and quantification of experimental joint inflammation in mice by measurement of 99mTc-pertechnetate uptake. Agents Actions.

[20] Robinson I, Sargent B, Hatcher JP (2012). Use of dynamic weight bearing as a novel end-point for the assessment of Freund’s Complete Adjuvant induced hypersensitivity in mice. Neurosci Lett.

[21] Dixon WJ (1991). Staircase bioassay: the up-and-down method. Neurosci Biobehav Rev.

[22] Chaplan SR, Bach FW, Pogrel JW, Chung JM, Yaksh TL (1994). Quantitative assessment of tactile allodynia in the rat paw. J Neurosci Methods.

[23] Carotti M, Salaffi F, Di Carlo M, Giovagnoni A (2017). Relationship between magnetic resonance imaging findings, radiological grading, psychological distress and pain in patients with symptomatic knee osteoarthritis. Radiol Med.

[24] Walsh DA, McWilliams DF (2012). Pain in rheumatoid arthritis. Curr Pain Headache Rep.

[25] Schelbergen RF, Geven EJ, van den Bosch MH, Eriksson H, Leanderson T, Vogl T (2015). Prophylactic treatment with S100A9 inhibitor paquinimod reduces pathology in experimental collagenase-induced osteoarthritis. Ann Rheum Dis.

[26] Sellam J, Berenbaum F (2010). The role of synovitis in pathophysiology and clinical symptoms of osteoarthritis. Nat Rev Rheumatol.

[27] Lees S, Golub SB, Last K, Zeng W, Jackson DC, Sutton P, Fosang AJ (2015). Bioactivity in an Aggrecan 32-mer fragment is mediated via Toll-like receptor 2. Arthritis Rheumatol.

[28] Miller RE, Ishihara S, Tran PB, Golub SB, Last K, Miller RJ et al. An aggrecan fragment drives osteoarthritis pain through Toll-like receptor 2. JCI Insight. 2018;3(6). 10.1172/jci.insight.95704. 10.1172/jci.insight.95704 PMC5926921 29563338

[29] Allette YM, Due MR, Wilson SM, Feldman P, Ripsch MS, Khanna R, White FA (2014). Identification of a functional interaction of HMGB1 with receptor for advanced glycation end-products in a model of neuropathic pain. Brain Behav Immun.

[30] McNamee KE, Alzabin S, Hughes JP, Anand P, Feldmann M, Williams RO (2011). IL-17 induces hyperalgesia via TNF-dependent neutrophil infiltration. Pain..

[31] Pitzer C, Kuner R, Tappe-Theodor A (2016). Voluntary and evoked behavioral correlates in inflammatory pain conditions under different social housing conditions. Pain Rep.

[32] Abu-Ghefreh AA, Masocha W (2010). Enhancement of antinociception by coadministration of minocycline and a non-steroidal anti-inflammatory drug indomethacin in naïve mice and murine models of LPS-induced thermal hyperalgesia and monoarthritis. BMC Musculoskelet Disord.

[33] Parvathy SS, Masocha W (2013). Gait analysis of C57BL/6 mice with complete Freund’s adjuvant-induced arthritis using the CatWalk system. BMC Musculoskelet Disord.

[34] Coulthard P, Pleuvry BJ, Brewster M, Wilson KL, Macfarlane TV (2002). Gait analysis as an objective measure in a chronic pain model. J Neurosci Methods.

[35] Hoffmann MH, Hopf R, Niederreiter B, Redl H, Smolen JS, Steiner G (2010). Gait changes precede overt arthritis and strongly correlate with symptoms and histopathological events in pristane-induced arthritis. Arthritis Res Ther.

[36] Malfait AM, Ritchie J, Gil AS, Austin JS, Hartke J, Qin W (2010). ADAMTS-5 deficient mice do not develop mechanical allodynia associated with osteoarthritis following medial meniscal destabilization. Osteoarthr Cartil.

[37] Bradman MJ, Ferrini F, Salio C, Merighi A (2015). Practical mechanical threshold estimation in rodents using von Frey hairs/Semmes-Weinstein monofilaments: towards a rational method. J Neurosci Methods.

[38] Guerrero AT, Pinto LG, Cunha FQ, Ferreira SH, Alves-Filho JC, Verri WA (2016). Mechanisms underlying the hyperalgesic responses triggered by joint activation of TLR4. Pharmacol Rep.

[39] Shibasaki M, Sasaki M, Miura M, Mizukoshi K, Ueno H, Hashimoto S (2010). Induction of high mobility group box-1 in dorsal root ganglion contributes to pain hypersensitivity after peripheral nerve injury. Pain..

[40] Kakigi R, Inui K, Tamura Y (2005). Electrophysiological studies on human pain perception. Clin Neurophysiol.

[41] Ferreira-Gomes J, Adães S, Sarkander J, Castro-Lopes JM (2010). Phenotypic alterations of neurons that innervate osteoarthritic joints in rats. Arthritis Rheum.

[42] Miller RE, Tran PB, Das R, Ghoreishi-Haack N, Ren D, Miller RJ, Malfait AM (2012). CCR2 chemokine receptor signaling mediates pain in experimental osteoarthritis. Proc Natl Acad Sci U S A.

[43] Miller RE, Tran PB, Ishihara S, Larkin J, Malfait AM (2016). Therapeutic effects of an anti-ADAMTS-5 antibody on joint damage and mechanical allodynia in a murine model of osteoarthritis. Osteoarthr Cartil.

[44] Illias AM, Gist AC, Zhang H, Kosturakis AK, Dougherty PM (2018). Chemokine CCL2 and its receptor CCR2 in the dorsal root ganglion contribute to oxaliplatin-induced mechanical hypersensitivity. Pain..

[45] Koenders MI, Kolls JK, Oppers-Walgreen B, van den Bersselaar L, Joosten LA, Schurr JR (2005). Interleukin-17 receptor deficiency results in impaired synovial expression of interleukin-1 and matrix metalloproteinases 3, 9, and 13 and prevents cartilage destruction during chronic reactivated streptococcal cell wall-induced arthritis. Arthritis Rheum.

[46] Ashraf S, Mapp PI, Shahtaheri SM, Walsh DA (2018). Effects of carrageenan induced synovitis on joint damage and pain in a rat model of knee osteoarthritis. Osteoarthr Cartil.

[47] Tse KH, Chow KB, Leung WK, Wong YH, Wise H (2014). Lipopolysaccharide differentially modulates expression of cytokines and cyclooxygenases in dorsal root ganglion cells via Toll-like receptor-4 dependent pathways. Neuroscience..

[48] Christianson CA, Corr M, Firestein GS, Mobargha A, Yaksh TL, Svensson CI (2010). Characterization of the acute and persistent pain state present in K/BxN serum transfer arthritis. Pain..

[49] Woller SA, Eddinger KA, Corr M, Yaksh TL (2017). An overview of pathways encoding nociception. Clin Exp Rheumatol.

[50] Schaible HG (2014). Nociceptive neurons detect cytokines in arthritis. Arthritis Res Ther..

[51] Massier J, Eitner A, Segond von Banchet G, Schaible HG (2015). Effects of differently activated rodent macrophages on sensory neurons: implications for arthritis pain. Arthritis Rheumatol..

[52] Woller SA, Ravula SB, Tucci FC, Beaton G, Corr M, Isseroff RR (2016). Systemic TAK-242 prevents intrathecal LPS evoked hyperalgesia in male, but not female mice and prevents delayed allodynia following intraplantar formalin in both male and female mice: the role of TLR4 in the evolution of a persistent pain state. Brain Behav Immun.

[53] Christianson CA, Dumlao DS, Stokes JA, Dennis EA, Svensson CI, Corr M (2011). Spinal TLR4 mediates the transition to a persistent mechanical hypersensitivity after the resolution of inflammation in serum-transferred arthritis. Pain..

[54] Bergman E, Johnson H, Zhang X, Hökfelt T, Ulfhake B (1996). Neuropeptides and neurotrophin receptor mRNAs in primary sensory neurons of aged rats. J Comp Neurol.

